# Operationalising MDT streamlining through standards of care: a framework for specification and implementation

**DOI:** 10.3389/frhs.2026.1794030

**Published:** 2026-06-03

**Authors:** Tayana Soukup, Wasim Hamad, Ellen Quinney, Benjamin W. Lamb

**Affiliations:** 1 Department of Surgery and Cancer, Faculty of Medicine, Imperial College London, London, United Kingdom; 2 Barts Cancer Institute, Queen Mary University of London, London, United Kingdom; 3 NHS Northeast London Cancer Alliance, London, United Kingdom; 4 Department of Urology, Barts Health NHS Trust, London, United Kingdom; 5 Department of Urology, University College London Hospitals NHS Foundation Trust, London, United Kingdom

**Keywords:** cancer services, case complexity, clinical governance, decision-making, MDT streamlining, multidisciplinary team meetings, service redesign, standards of care

## Abstract

Multidisciplinary team meetings (MDTMs) are central to cancer treatment planning in the UK, but increasing caseloads, growing clinical complexity, and workforce constraints have raised concerns about the sustainability of a “discuss-every-case” model. National guidance in England now promotes MDT streamlining, using Standards of Care (SoCs) to stratify cases requiring full multidisciplinary discussion from those suitable for standardised pathways. However, there remains limited clarity on how SoCs should be specified and governed to ensure safety, consistency, and clinical accountability in routine practice. This mini-review synthesises national policy, emerging empirical literature on MDT streamlining, and evidence from complexity research to examine SoCs as a mechanism for operationalising MDT reform. We highlight that empirical evaluations of streamlining remain limited and heterogeneous, and that reported effects depend less on caseload reduction than on information readiness, organisational context, and explicit escalation criteria. Drawing on NHS England guidance, specialty recommendations from the British Association of Urological Surgeons (BAUS), and the Measure of case-Discussion Complexity (MeDiC), we present a structured framework to support SoC development. The framework specifies parameters across patient, pathology, and treatment domains (in line with the MeDiC tool), alongside explicit eligibility, exception, and escalation logic, and interfaces with governance requirements for triage, data completeness, and audit. By making decision boundaries transparent and multidimensional, this approach addresses key risks associated with oversimplification and inappropriate exclusion while preserving clinical judgement and patient-centred care. Structured approaches to SoC development provide a defensible foundation for focusing MDT discussion where it adds greatest value under increasing service pressures.

## 1 Introduction

Multidisciplinary team (MDT) meetings (MDTMs) have been widely adopted as the “gold standard” approach to cancer treatment planning in the UK, reflecting a long-standing policy commitment to coordinated, specialist-led decision-making following the Calman–Hine reforms and subsequent national guidance on cancer services (1–4). In principle, MDTMs provide a structured forum in which clinically diverse professionals integrate diagnostic, pathological, and therapeutic information to formulate consistent, evidence-informed recommendations for patients with suspected or confirmed cancer. However, the effectiveness of MDT working depends not simply on the existence of MDTMs, but on how these meetings function in practice: how information is presented, how teams interact, and how decisions are reached and implemented (5–8).

Our synthesis of MDT implementation evidence and subsequent observational research have demonstrated that MDTMs are best understood as complex sociotechnical decision environments rather than simple case-review forums. MDT effectiveness is shaped by modifiable factors including leadership and meeting management, information availability and quality, team participation, workload, and logistical constraints (5–8). In a large prospective observational study of MDT decision-making across 822 cancer cases, we identified systematic relationships between time pressure, workload, task complexity, logistical challenges, group composition, and communication and interaction patterns, indicating that MDT performance is sensitive to both human and system-level conditions (6, 7). Further work showed that logistical problems during MDTMs are associated with poorer communication and decision-making, reinforcing that “how the meeting runs” can materially influence the quality of multidisciplinary discussion, and by inference, downstream patient care (8).

These findings are particularly salient in the context of contemporary cancer services, which face sustained growth in demand and complexity. Increasing caseloads, expanding diagnostic and treatment options, and persistent workforce constraints have placed growing demands on MDTMs, contributing to a mismatch between what they are expected to deliver and the time and cognitive capacity available. This may, in part, reflect the success of the MDT model itself, with MDTMs increasingly relied upon for decisions that may previously have been made independently or through informal peer consultation. While MDT working remains essential for complex and uncertain cases, the expectation that every case should receive full MDT discussion has become increasingly difficult to sustain under escalating workload pressures (2–4, 9–13).

This body of evidence supports a clear conclusion: improving MDT effectiveness cannot rely solely on incremental optimisation of meeting conduct. It also requires structural redesign that protects multidisciplinary discussion for the patients who most need it. This shift—from universal full discussion toward a stratified, value-based allocation of MDT time—has become central to recent national guidance and reform efforts (2–4).

## 2 MDT streamlining in policy and practice: from national guidance to renewed calls for reform

In England, MDT streamlining was formally articulated in NHS England and NHS Improvement guidance published in January 2020, which reframed MDTMs as a finite resource that should be focused on cases requiring full multidisciplinary input (2). The guidance established a central principle: all patients remain listed and recorded at MDTMs, but cases are stratified into those requiring full discussion and those whose needs can be met through an agreed Standard of Care (SoC) pathway and therefore do not require full, in-depth discussion at the meeting. The intent was not to remove MDT oversight, but to increase MDT capacity for cases where multidisciplinary discussion adds greatest value, while preserving transparency and consistency of pathways (2).

More recent literature has reinforced this policy direction. We and others have argued that streamlining represents a fundamental shift in MDT practice, requiring a move away from universal full discussion toward proportionate use of MDT time, while cautioning that poorly governed streamlining risks recreating unwarranted variation and undermining the original purpose of MDT working (14–16). These arguments emphasise the need for evidence-based, implementation-aware approaches rather than *ad hoc* caseload reduction (17).

Despite broad policy and professional consensus, implementation of streamlining after 2020 was slow and uneven. Publication of the guidance coincided with the onset of the COVID-19 pandemic, constraining service redesign capacity and contributing to variable uptake across MDTs and Cancer Alliances. As a result, MDTs have faced sustained workload pressure without access to consistently applied, governance-ready mechanisms for operationalising streamlining in practice.

As reform efforts resumed, attention increasingly converged on two interrelated challenges: how to identify and prioritise genuinely complex cases for MDT discussion, and how to establish safe, auditable pathways for non-complex cases that do not require full MDTM time. These issues underpin renewed reform initiatives, including the 2025 multi-society briefing led by the Royal College of Radiologists, which calls for substantive MDT reform and emphasises that MDTMs should focus on patients for whom multidisciplinary discussion is most likely to improve care. The briefing distinguishes MDT working from MDT meetings, highlighting the need for high-quality pathways and protocols to support consistent decisions for the majority of patients (3, 4).

Empirical evaluation of MDT streamlining remains limited. Only a small number of audit and observational studies have directly assessed streamlining within MDTMs, primarily in lung, gynaecological, and colorectal cancer. These studies suggest that streamlining can reduce repeated case discussion and enable more focused discussion on complex cases, with some evidence of increased discussion time per complex case, although consistent reductions in overall meeting duration have not been demonstrated. Where structured pathways or standards of care (SoCs) have been introduced, their effectiveness appears to be driven primarily by information completeness, organisational readiness, and clear eligibility and escalation criteria, rather than by caseload reduction alone (18–22). A broader body of observational (6–8), systematic reviews (23) and survey-based research (24–26) supports streamlining in principle as a response to workload pressures, while highlighting persistent concerns about patient safety, inappropriate exclusion, and erosion of clinical judgement when governance and escalation routes are insufficiently explicit.

This post-2020 literature highlights a persistent gap: while MDT streamlining is increasingly endorsed as necessary, there remains limited clarity on how streamlining through SoCs—should be specified, governed, and audited to ensure safety, scalability, and consistency across tumour pathways and organisational contexts. Addressing this gap provides the rationale for the framework presented in the sections that follow.

## 3 Standards of care as a mechanism for MDT streamlining

In the NHS England streamlining model, SoCs are the central mechanism through which MDT streamlining is operationalised. Streamlining is defined not as the removal of MDT oversight, but as the routine use of SoCs within MDT working to stratify cases into those requiring full multidisciplinary discussion and those that can be listed but not discussed in depth because their pathway needs are met by a predefined standard (2).

NHS England defines a SoC as a point in the patient pathway where there is consensus on a recognised international, national, regional, or local guideline regarding the intervention(s) that should be made available to a patient. Multiple SoCs may exist for a given disease stage or clinical scenario (including “watch and wait” approaches), but they are intended to apply only at points where there is clear agreement about management. In the national model, SoCs are developed by tumour-site specialist MDTs in collaboration with Cancer Alliance and are formally approved through Expert Reference Groups to support consistent application across an Alliance geography (2).

Conceptually, SoCs underpin streamlining by enabling proportionate MDT discussion, improving consistency and transparency, and preserving MDT capacity for complex cases. By reducing repeated discussion of predictable, low-risk presentations that meet clearly defined criteria, SoCs release MDTM time for cases where multidisciplinary input is most likely to change or clarify management. At the same time, explicit specification of eligibility criteria and escalation-triggers minimise unwarranted variation, which might be introduced through *ad hoc* case selection, and move MDTMs away from predominantly transactional activity, toward discussions focused on areas of uncertainty, competing risks, or complicating factors (2).

A defining feature of the NHS England approach is that SoCs do not replace clinical judgement. Rather, they provide a default pathway, anchored in guidelines and consensus, accompanied by explicit safeguards. All patients remain listed at MDTMs, application of an SoC must be supported by MDT members. Escalation to full discussion is always permissible where there is doubt, new information, patient preference, or physiological or psychosocial need (2).

Specialty-level guidance reinforces this paradigm. BAUS oncology guidance (27) describes how SoCs can be selected and operationalised in urological cancer pathways, emphasising that SoC-based streamlining, when applied to low-risk conditions with strong consensus for management, enables more meaningful discussion of genuinely complex cases. BAUS further highlights the importance of explicit exclusion criteria—such as frailty, comorbidity, psychosocial complexity, competing health issues, or misalignment with patient wishes—that mandate escalation to full MDT discussion, reinforcing that SoCs are governed standards with defined exception handling rather than rigid protocols (27).

Finally, SoCs align closely with the evidence-based framing of case complexity provided by the Measure of case-Discussion Complexity (MeDiC) tool—the first instrument designed to capture both clinical and logistical contributors to complexity—supporting safe selection and prioritisation of cases for multidisciplinary discussion (28). In this sense, SoCs and the MeDiC tool are complementary: SoCs define where care can be standardised with appropriate governance, while MeDiC clarifies which patient, pathology, treatment, or contextual features should trigger initial listing for, or escalation back into, full MDT discussion.

Together, BAUS oncology guidance and the MeDiC tool establish the clinical and complexity-based rationale for the Standards of Care development framework outlined in the next section.

The current evidence base informing MDT streamlining comprises a combination of empirical studies, conceptual work, and policy guidance, with relatively limited direct evaluation of Standards of Care (SoC) implementation. A summary of the relevant literature is presented in Table 1.

**Table 1:** Summary of empirical, conceptual, and policy literature informing MDT streamlining and standards of care (SoCs).

Study (Ref)	Study type	Publication type	Setting	Focus	Key findings
A. Empirical evaluation of MDT streamlining/SoCs					
Gore (18)	Service transformation report	Policy/report	NHS cancer services	MDT streamlining	Described inefficiencies in MDT processes and the need to reduce discussion of low-complexity cases
Zasada et al. (19, 20)	Observational study	Conference abstracts	Lung cancer MDTs	MDT streamlining	Reported potential to reduce repeated case discussion and support prioritisation of complex cases, with variable impact on meeting duration
Bansal et al. (21)	Audit	Conference abstract	Endometrial cancer MDT	SoC implementation	Suggested that implementation of SoCs may enable exclusion of low-risk cases from full MDT discussion
Davey et al. (22)	Observational study	Conference abstract	Colorectal MDTs	MDT efficiency	Suggested opportunities to optimise MDT discussions through case selection and prioritisation
B. Conceptual and strategy-focused literature					
Al Hammouri et al. (14)	Mini-review	Journal article	Multiple tumour types	Streamlining implementation	Summarised implementation approaches, highlighting need for structured triage, governance, and standardisation through SoCs
Soukup et al. (15)	Narrative review	Journal article	MDTs broadly	Streamlining challenges	Highlighted challenges of streamlining, including need for explicit eligibility, escalation, and audit processes
Winters et al. (16)	Commentary	Journal article	MDTs broadly	MDT reform	Argued that increasing workload and complexity require a shift away from universal case discussion
Soukup et al. (17)	Conceptual paper	Journal article	MDTs broadly	Streamlining strategy	Proposed a strategy for prioritising complex cases within MDT workflows
C. Guidelines and policy frameworks					
NHS England (2)	National guidance	Policy/report	NHS cancer services	MDT streamlining/SoCs	Defined SoCs as the mechanism for streamlining and specified requirements for triage, governance, and audit
Lamb et al. (27)	Specialty guidance	Guideline	Urological cancer MDTs	SoC operationalisation	Provided guidance on selecting SoCs, defining eligibility, exclusion, and escalation criteria
D. Clinician perspectives and acceptability					
Hoinville et al. (24)	Survey study	Journal article	UK MDT members	Perceptions of streamlining	Reported support for streamlining alongside concerns regarding safety and decision-making
Jalil et al. (25)	Survey	Conference abstract	Urology MDTs	Streamlining acceptability	Reported mixed clinician perspectives, incL. concerns about inappropriate case exclusion
Warner et al. (26)	Comparative observational study	Journal article	Urology and other MDTs	MDT restructuring	Supported refocusing MDT discussions on complex cases requiring multidisciplinary input
E. Operationalisation of case selection					
Soukup et al. (28)	Mixed-methods study (tool development and validation)	Journal article	Multiple cancer MDTs	Case complexity assessment (MeDiC tool)	Developed and validated a structured measure of case complexity to support consistent prioritisation and escalation of cases for MDT discussion

## 4 A framework for developing standards of care to support MDT streamlining

While national guidance clearly positions SoCs as the mechanism for MDT streamlining, it is less explicit about how MDTs and Cancer Alliances should translate guideline recommendations into SoCs that are operationally usable, governance-ready, and consistently applied across sites. In our Cancer Alliance streamlining programme, SoC development emerged as a critical first step because it requires interdisciplinary, alliance wide, agreement on what constitutes a “straightforward” pathway decision, what minimum information is required to apply that decision safely, and what conditions mandate escalation to full MDT discussion. At a system level, this focus aligns with the NHS England implementation model, which places Cancer Alliances at the centre of coordinating SoC development, securing pathway board approval, supporting Trust-level clinical governance processes, agreeing triage roles and processes, and embedding audit prior to wider rollout (2).

**Figure 1:**
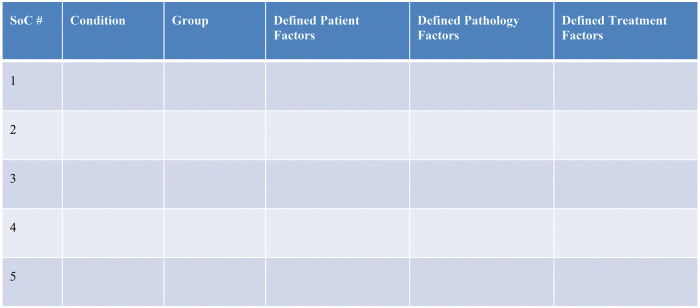
Framework for determining clinical parameters for standards of care (SoCs) to support MDT streamlining.

To support this interdisciplinary task, we developed a framework for SoC development (Figure 1). The framework was designed to facilitate rapid shared understanding across clinical and operational stakeholders and to ensure that SoCs are specified in a manner that aligns with national governance requirements and routine MDT workflow. It was developed by synthesising three complementary sources: (i) NHS England guidance on the definition, governance, and audit of SoCs within MDT streamlining; (ii) BAUS oncology streamlining guidance, which provides pragmatic specialty-level considerations for SoC selection and triage models; and (iii) the MeDiC tool, which offers an evidence-based structure for conceptualising case complexity across clinical and logistical domains and therefore supports defensible escalation criteria (2, 27, 28).

### 4.1 The core components of the framework

**Figure 2:**
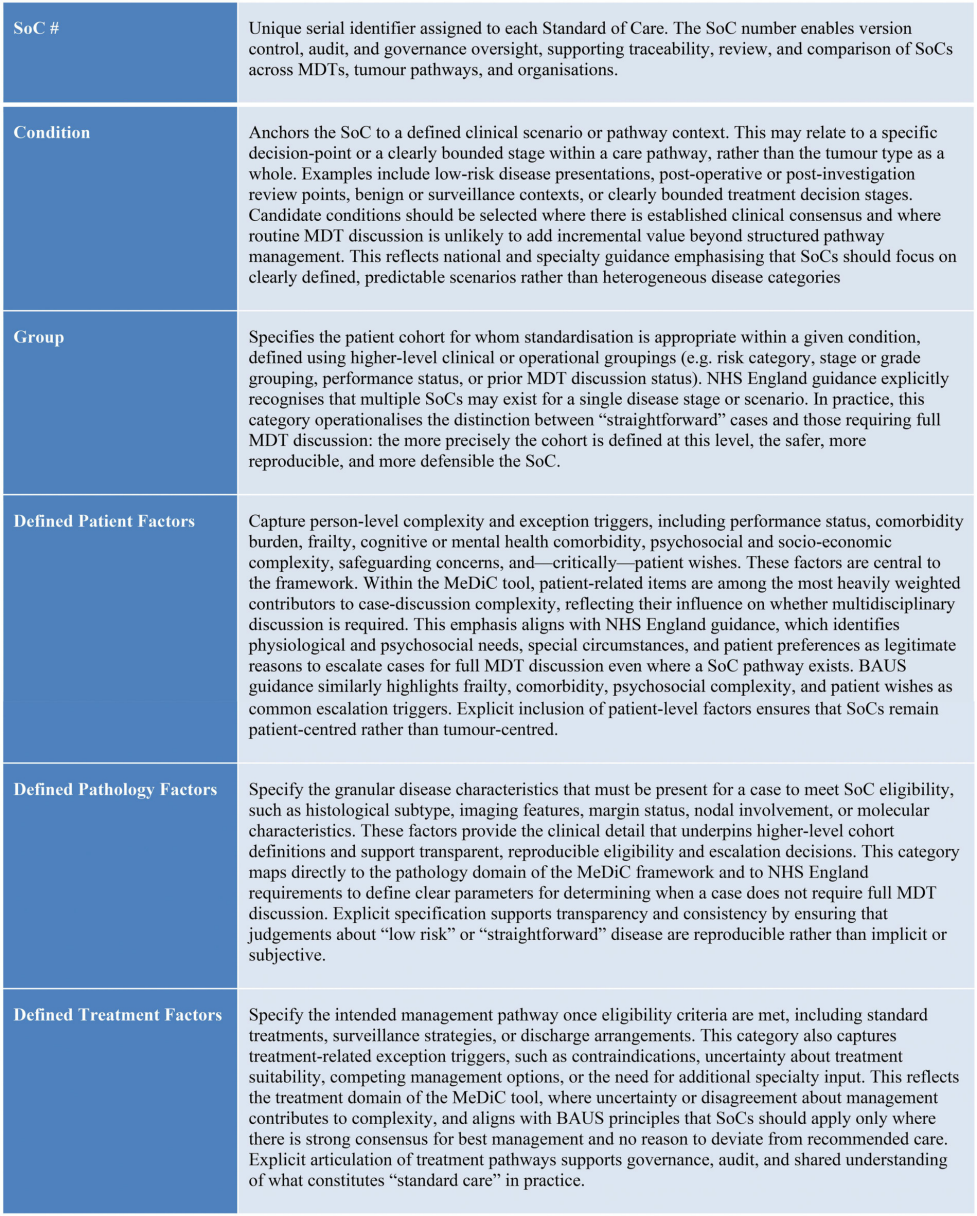
Definitions of framework categories used to specify standards of care (SoCs) for MDT streamlining.

The framework is structured as a tabular template comprising five core fields—Condition, Group, Defined Patient Factors, Defined Pathology Factors, and Defined Treatment Factors—each of which contributes to explicit specification of eligibility, standardisation, and escalation logic for individual SoCs (Figure 1). Definitions and scope of each framework category are provided in Figure 2.

### 4.2 Operationalising safe streamlining

The central contribution of the framework is that it makes SoC logic explicit across the three domains that MDT decision-making consistently depends on—patient, pathology, and treatment—rather than allowing SoCs to become overly narrow (e.g., pathology-only) or overly broad (e.g., “routine cases”). This design reflects evidence from the MeDiC tool that case complexity is multidimensional and aligns with NHS England guidance specifying that patient circumstances and preferences must be considered even when streamlined pathways are used (2, 28).

In practice, the framework supports safe and scalable streamlining by separating three elements that are often conflated in MDT discussions: agreement on the standard management recommendation, agreement on eligibility criteria for applying that standard, and agreement on exception triggers requiring escalation to full MDT discussion. This separation enables more efficient consensus-building while preserving explicit routes for clinical judgement, patient-centred exception handling, and escalation, thereby reducing unwarranted variability in routine cases without constraining decision-making where complexity is present (2, 27, 28).

### 4.3 Scope and intended use

The framework is intended as a developmental and consensus-support tool for Cancer Alliances and MDTs engaged in streamlining, rather than as a prescriptive model for SoC content or MDT operation. Its purpose is to support the structured translation of existing guideline consensus into SoCs that are sufficiently explicit to be operationalised and sufficiently robust to support governance, audit, and review. Consistent with NHS England guidance, the framework is designed to enable a principle of “standard where appropriate; discuss where needed”, supported by clearly articulated eligibility criteria, escalation routes, and mechanisms for ongoing refinement through audit (2).

### 4.4 Minimum dataset and conditions required for SoC implementation

While the framework presented in Figure 1 specifies the clinical parameters that define a SoC, NHS England guidance (2) makes clear that SoCs can only be applied safely when supported by defined operational conditions. Accordingly, the framework interfaces directly with requirements relating to information readiness, triage processes, and governance, clarifying what is required for a SoC to be implementation-ready.

A core requirement is the availability of a minimum dataset before a patient can be assigned to a “not for full discussion” streamlined pathway. NHS England specifies that this includes confirmation of diagnosis and staging, designated reporting of imaging and pathology, assessment of fitness and comorbidities, documentation of patient preferences and special circumstances, and completion of relevant investigations (2). Embedding these requirements within SoC design ensures that streamlining decisions are made only when information completeness is sufficient to support safe, transparent, and accountable decision-making.

SoC implementation also depends on agreed triage roles, responsibilities, and workflows. NHS England requires Trust Medical Director-level agreement of a pre-MDTM triage process, with clarity regarding who assigns cases to SoCs and who retains clinical oversight (2). BAUS guidance describes several viable triage models, including mini-MDT approaches, lead-and-coordinator triage, and distributed clinician-led triage with real-time oversight and sign-off by a core MDT member (27). Even well-specified SoCs will fail if triage processes are ambiguous, under-resourced, or insufficiently embedded within routine workflows.

Finally, NHS England positions audit and review as essential conditions for safe streamlining. Audit is expected to be embedded before SoCs go live, with MDTs reviewing a sample of cases on a quarterly basis, including both patients managed under SoCs and those for full discussion (2). This positions SoCs as dynamic standards rather than static protocols, enabling cases assigned to an SoC to be refined, expanded, or escalated back into full MDT discussion in response to audit findings, service change, or emerging evidence, while preserving transparency and professional accountability.

Together, these conditions reinforce that the framework is conceptual and translational rather than prescriptive. It does not replace tumour-site guidelines or local governance arrangements; instead, it provides a structured method for translating guideline consensus into SoCs that can be implemented safely within existing MDT governance structures. Consistent with national guidance and BAUS principles, clinical discretion is preserved by design, with explicit routes for escalation back to full MDT discussion when patient preference, new information, or complexity warrants it (2, 27).

## 5 Discussion: design considerations and challenges in implementing standards of care

Although Standards of Care (SoCs) are positioned as the central mechanism for operationalising MDT streamlining while preserving safety, consistency, and professional accountability, their introduction represents a substantive redesign of MDT working rather than a simple technical adjustment. Their success therefore depends on careful attention to design boundaries, governance, professional ownership, and implementation context. Drawing on national guidance, the emerging streamlining literature, and our experience developing a framework to support SoC specification, we highlight key considerations and risks that must be addressed if SoCs are to deliver their intended benefits.

### 5.1 Defining boundaries and avoiding inappropriate exclusion

A primary design challenge concerns where SoCs should—and should not—be applied. Both NHS England guidance (2) and specialty-level recommendations (27) emphasise that SoCs are most appropriate for high-volume, low-risk scenarios characterised by strong clinical consensus. Extending SoCs into areas marked by uncertainty, competing treatment options, or preference sensitivity risks undermining the rationale for multidisciplinary discussion itself.

Poorly defined scope can lead to two opposing but equally problematic outcomes: SoCs that are too narrow to release MDT capacity, or SoCs that are too broad and inappropriately divert cases from MDT discussion. A central safety risk is misclassification, whereby cases that appear straightforward based on tumour characteristics alone may nonetheless warrant MDT input due to patient-level, treatment-level, or contextual complexity (27, 28). In such circumstances, oversimplified or poorly specified SoCs risk excluding patients whose needs extend beyond standard pathways and, paradoxically, increase reliance on additional clinical discretion and decision-making, running counter to the ethos of SoCs.

The framework presented in this review addresses this risk by requiring explicit specification of eligibility and exclusion criteria across patient, pathology, and treatment domains. By making these boundaries transparent and contestable, rather than implicit, the framework supports safer application of SoCs, reducing the likelihood of inappropriate exclusion or inclusion.

### 5.2 Governance, continuous audit, and adaptability as safety infrastructure

SoCs are only as safe as the governance structures that support them. NHS England guidance is explicit that SoCs must be embedded within formal governance arrangements, including Alliance-level agreement, Trust clinical sign-off, pre-MDTM triage processes, and routine audit. These requirements should be understood not as administrative overheads, but as essential safety infrastructure (2).

Audit plays a particularly critical role by providing assurance that SoCs are applied appropriately and creating a mechanism for learning and adaptation. Without systematic review, SoCs risk becoming static artefacts that drift from current evidence or local service realities. In contrast, when audit findings are used to refine, expand, or withdraw SoCs, streamlining becomes a dynamic process aligned with continuous improvement rather than a one-off service redesign (2).

Adaptability is therefore a defining feature of safe streamlining. As evidence evolves, technologies change, and service configurations shift, SoCs must be reviewed and updated to maintain clinician confidence and patient safety. This adaptive capacity also supports scalability: MDTs can progressively develop additional SoCs where appropriate, while modifying or retiring standards that do not perform as intended (2).

### 5.3 MDT ownership, professional trust, and operational feasibility

A frequently cited concern in streamlining initiatives is the potential erosion of professional autonomy and trust. If SoCs are perceived as externally imposed rules or cost-containment tools, clinicians may disengage or create informal workarounds that undermine transparency and governance (24–26). This risk is particularly salient in MDTs, where legitimacy is closely tied to collective discussion and shared responsibility.

Both national (2) and specialty guidance (27) emphasise that SoCs should be clinically led and co-produced, with explicit recognition that escalation to full MDT discussion is always permissible. Framing SoCs as a shared articulation of “what we already agree on”, that is, as MDT decisions reached through prior discussion and agreement, rather than as restrictions on judgement is critical for preserving MDT ownership. The explicit incorporation of exception handling and patient preference within SoC logic further reinforces that streamlining is intended to support, not replace, clinical and MDT decision-making.

Operational feasibility is equally important. SoCs depend on timely access to reliable information and clearly defined triage roles. Standards that rely on data that are inconsistently available, or on triage responsibilities that are poorly resourced, are unlikely to be applied consistently and sustainably in practice. Considering operational requirements at the design stage—rather than retrospectively—is therefore essential to avoid SoCs becoming “paper standards” that exist in principle but not in routine MDT workflow.

### 5.4 Evidence gaps and implications for future work

Despite growing policy and professional consensus on the need for MDT streamlining, the empirical evidence base evaluating SoC-based approaches remains limited. Published studies describe heterogeneous implementations, variable outcome measures, and limited assessment of unintended consequences (18–22). As a result, many current design decisions rely on informed anticipation rather than robust comparative evidence. The framework presented in this mini-review addresses a specific gap by offering a structured approach to SoC development grounded in national guidance and research. However, further empirical work is needed to evaluate how such frameworks perform in practice, including their impact on MDT workload, decision quality, equity, and patient outcomes. Future studies should examine not only efficiency gains but also safety, acceptability, and sustainability over time.

## 6 Conclusion

SoCs provide a governance-ready mechanism for operationalising MDT streamlining that preserves clinical judgement, patient safety, and multidisciplinary accountability. By making eligibility, escalation, and decision boundaries explicit across patient, pathology, and treatment domains, structured approaches to SoC development offer a defensible foundation for focusing MDT discussion where it adds greatest value in increasingly pressured cancer services.
